# Challenges in turning a great idea into great health policy: the case of integrated care

**DOI:** 10.1186/s12913-020-4950-z

**Published:** 2020-02-21

**Authors:** Kasper Raus, Eric Mortier, Kristof Eeckloo

**Affiliations:** Ghent University Hospital and Ghent University, Corneel Heymanslaan 10, 9000 Ghent, Belgium

**Keywords:** Integrated care, Health policy, Collaboration

## Abstract

**Background:**

In the organization of health care and health care systems, there is an increasing trend towards integrated care. Policy-makers from different countries are creating policies intended to promote cooperation and collaboration between health care providers, while facilitating the integration of different health care services. Hopes are high, as such collaboration and integration of care are believed to save resources and improve quality. However, policy-makers are likely to encounter various challenges and limitations when attempting to turn these great ideas into effective policies. In this paper, we look into these challenges.

**Main body:**

We argue that the organization of health care and integrated care is of public concern, and should thus be of crucial interest to policy-makers. We highlight three challenges or limitations likely to be encountered by policy-makers in integrated care. These are: (1) conceptual challenges; (2) empirical/methodological challenges; and (3) resource challenges. We will argue that it is still unclear what integrated care means and how we should measure it. ‘Integrated care’ is a single label that can refer to a great number of different processes. It can describe the integration of care for individual patients, the integration of services aimed at particular patient groups or particular conditions, or it can refer to institution-wide collaborations between different health care providers. We subsequently argue that health reform inevitably possesses a political context that should be taken into account. We also show how evidence supporting integrated care may not guarantee success in every context. Finally, we will discuss how promoting collaboration and integration might actually demand more resources. In the final section, we look at three different paradigmatic examples of integrated care policy: Norway, the UK’s NHS, and Belgium.

**Conclusions:**

There seems widespread agreement that collaboration and integration are the way forward for health care and health care systems. Nevertheless, we argue that policy-makers should remain careful; they should carefully consider what they hope to achieve, the amount of resources they are willing to invest, and how they will evaluate the success of their policy.

## Background

There is no denying that health care systems worldwide are complex systems [1]. One of the most important changes in contemporary health care systems worldwide is the trend towards interorganizational collaboration for integrated care [2–5]. Whereas individual health care institutions have traditionally been seen as the main focal points of health care systems, there is growing recognition that individual patients and communities have health needs that often require the coordinated involvement of more than one medical speciality or health institution.

There seem to be two important drivers. First, there is an important economic driver: interorganizational collaboration may be an attractive way for institutions to reduce costs and consolidate market share [6]. Health care institutions that offer particular, though different, services in a care trajectory could, for example, come together to collaborate so as to offer patients a full care trajectory. Second, interorganizational collaboration is often driven by necessity, because of changes in demographics. Research in many countries indicates an increase in both elderly patients and in multimorbidity, and these trends are expected to continue into the future [7]. A recent study, for example, found that the number of elderly patients in England with four or more medical conditions is likely to double between 2015 and 2035 [8]. Such patients most often require treatment from various different specialists, and treatment for one condition often interferes with the treatment for another. One excellent study into multimorbidity charts the medical trajectory of the average elderly diabetes patients to show the enormous complexity of their care trajectory, which annually involves around 80 health-related services and interactions with seven or eight different health professionals [9]. A change in the classic healthcare system is becoming imperative.

In this paper, we focus on formulating and implementing of policies that mandate, allowing, or encouraging interorganizational collaborations for integrated care. This is now high on the policy agenda in many countries [10], and many programmes have been set up [11, 12]. This is no surprise, as such programmes have been argued to potentially improve patient care and to help more efficiently allocate scarce resources [13, 14].

However, while there is a certain amount of agreement that integration of care is a good thing, drafting effective policy on integrated care proves to be more difficult. We will therefore highlight what we identify as the challenges or limitations that policy-makers face when drafting policy on integrated care. First, we consider how the drafting and implementation of policy in integrated care are likely to face *conceptual challenges*. Second, there is an important *methodological–empirical challenge* for policy-makers attempting to base their policy on existing evidence. Third and finally, we highlight some *resource challenges*.

In the final part of our paper, we briefly look at three different examples of policy on integrated care: namely, policy in Norway, the UK, and Belgium.

## Main text

### Policy, politics, and health

Interorganizational collaboration for integrated care can come about in a variety of ways—for example, through market mechanisms or health care institutions coming together around particular values [15]. In this paper, we focus on the role of policy, as we believe that at least some involvement of policy-makers and political commitment is required. Health and health care are by nature profoundly political, and thus require political action for their organization [16, 17]. As argued by Bambra et al. 2005 [16], there are three reasons this is the case:

- Health is unequally distributed in society, and some individuals or groups have more of it than others
- Health has social determinants that are amenable to political interventions. Changing people’s health for the better requires the involvement of more than the health care sector
- Decent standards of living and well-being are essential for full citizenship and are recognized as human rights

Governments and policy-makers have a clear duty to make sure that health is at all times distributed fairly and that the health care system is organized in a just way. If there is a shift towards interorganizational collaboration, this itself justifies health policy involvement to make sure the shift promotes fairness. As summarized in the classic paper by Campbell (1969), *removing reform administrators from the political spotlight seems both highly unlikely, and undesirable even if it were possible* [18: 409].

It is important to be clear that, in this paper we are focusing on the challenges that arise when *drafting* policy on interorganizational collaboration. Naturally, there are also challenges related to the successful implementation of such policy in practice. However, there are two important reasons for not fully exploring this topic here: First, providing sufficient attention to this topic would merit a paper of its own, as there are a number of different ways to implement policy. Classically, we could distinguish coercive top-down implementation (e.g., where policy-makers simply mandate a particular course of action) and more bottom-up implementations (such as where policy-makers create the right circumstances to encourage professionals or institutions to voluntarily work together in a more integrative way). A third strategy is the consultative approach, where policy-makers (for example) consult particular interest groups or expert panels in order to make a decision. Such a strategy lies somewhere between because, while it is the policy-maker who finally decides (in a top-down fashion), there is also involvement from relevant policy stakeholders (bottom-up). Finally, there is the possibility that policy-makers themselves are mandated to devise or revise policy, for example by supranational institutions or policy-makers. In the case of integrated care, it is clear that a European Regulation such as the General Data Protection Regulation (GDPR) will have great impact on how and when data can be shared, and thus (directly or indirectly) on how integrated care can be organized. Each of these strategies involves particular challenges which we cannot fully explore in the scope of this particular paper.

Secondly, we believe that drafting good policy is crucial, and that the difference between policy drafting and policy implementation should not be overstated. A policy that was not implemented successfully because of circumstances that were foreseeable to policy-makers was never good policy to start with, since it was clearly not suited to the circumstances. Our paper focuses on the challenges policy-makers are likely to meet, and will have to meet in order to make sure their policy has the best possible chance of being successfully implemented.

### Potential challenges and limitations

#### Conceptual challenges

Although collaboration and integrated care are often praised, there remains a significant lack of clarity on what constitutes integrated care [18–20]. The concepts of ‘integrated care’ and ‘interorganizational collaboration’ could be considered catch-all terms. When drafting and implementing integrated care, there are three fundamental questions that should be considered by every policy-maker: (1) how the integration will be organized; (2) what kind of integration is intended; (3) what outcome is intended.

How will the integration be organized? There is no doubt that integration is not an all-or-nothing concept, but instead comes in a wide variety of degrees. Two institutions could, for example, remain fully autonomous entities that merely coordinate the health care services they provide so that patients can have a smooth care trajectory. On the other end of the spectrum, they might also come to a more formal and structured collaboration (such as through joint governance structures) or even full integration (like through mergers or contracts) [18, 21]. Integrated care thus has a vagueness that can and should be translated into more concrete governance forms, if it is to be successfully implemented.

What kind of integration is intended? This is key, as integrated care has been argued to have many dimensions. A common distinction is between (1) clinical integration, (2) professional integration, (3) organizational integration (collaborations between organizations), (4) systemic integration (integration at the health system level), (5) functional integration (the communication of data and information within the integrated care system), and (6) normative integration (see Table 1) [19, 22]. Acknowledging the distinctions between these dimensions is crucial since different people talking about ‘integrated care’ might actually be talking about very different kinds of integration.

**Table 1:** Types of integration

	Types of integration
Micro level	1. Clinical integration
Meso level	2. Professional integration 3. Organizational integration
Macro level	4. Systemic integration
Possible at all levels	5. Functional integration 6. Normative integration

What outcome is intended? It is evident that there may be different reasons why integration is desired. These could include individual health-related outcomes (such as improved quality or patient experiences), outcomes related to population health (like fewer unnecessary transfers and fewer unnecessary health care interventions in general), financial outcomes (e.g., reducing the global cost of the health care system), and even social/ethical outcomes (such as improving fairness in allocating medical resources). The Triple Aim model of integrated care, as formulated by the American Institute of Health Care Improvement (IHI), believes that integrated care should aim to (1) improve the individual experience of care; (2) improve the health of populations; and (3) ‘reduce the per capita cost of health care’ [23]. More recently, several commentator have argued that the Triple Aim model ought to be replaced by the Quadruple Aim model, which adds the fourth aim of improving health care providers’ work life (see Table 2) [24, 25]. However, it is possible that a particular policy might not achieve each possible outcome; a political choice must then be made and some kind of balance must be struck. Integrated care by no means involves a single ideology that is shared by all who promote it.

**Table 2:**
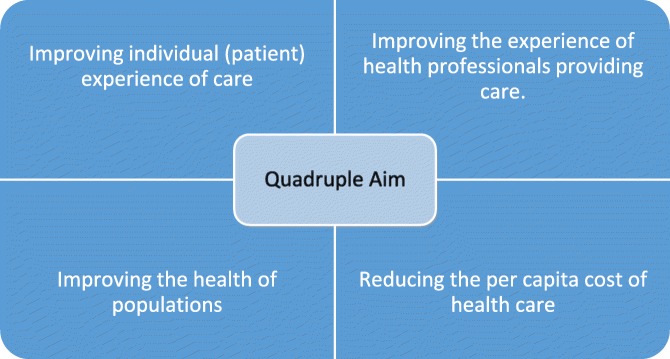
Quadruple Aim

Because of the conceptual complexity and ideological choices, it is particularly difficult to determine when and to what degree integration of care is a success. In 2009, a systematic review identified 24 different methods of measuring integrated care delivery [26]. In 2017, a new systematic review found 114 unique tools for measuring health system integration across a total of sixteen domains [27]. Conceptual confusion seems only to have increased. Nevertheless, distinguishing between the organization of the integrated care network (domain 1), the kind of integration that is aimed for (domain 2), and the integration outcome that is aimed for (domain 3) remains crucial. Failure to do so when evaluating a policy can lead to misleading results. For example, one might find a high level of trust between partners in an integrated care network (domain 1), but this need not imply a high level of integration (domain 2) or of beneficial outcomes (domain 3). Likewise, although there may be tools to evaluate the level of integration within organizations and between organizations (domain 2), this need not always translate into better outcomes (domain 3). One striking example is the recent cluster-randomized trial of a more integrative 3D approach to treating multimorbidity in primary care [28]. Patients here received a six-monthly whole-person review that involved reviews by a nurse, pharmacist, and physician. The study found that, although clinical integration improved, there was no evidence for the 3D approach actually improving patients’ quality of life.

Amidst all the confusion, we must of course acknowledge that there have been attempts to find a conceptual and all-encompassing theoretical framework. One well-known model is the Rainbow model [29], which expands on the classic Triple Aim Model [29]. Besides the three classic aims, the Rainbow model introduces two care-guiding principles (population-based care and person-focused care) and six domains of integrated care (clinical, professional, organizational, system, functional, and normative integration). Together these result in a complex rainbow of different kinds of integration. Currently there are studies underway that attempt to validate an integration measurement tool based on this Rainbow Model [30]. Time will tell whether this model can function as a gold standard, but we believe that much remains to be done. For one, the Rainbow model was developed for the primary care setting, and is thus mainly focused on horizontal integration. To operate in a complete and complex health care system with both horizontal integration (e.g. when health care organizations collaborate or merge) and vertical integration (e.g. collaborations between primary, secondary, and tertiary settings), the model might need to be expanded.

#### Empirical challenges

In drafting policy on integrated care, policy-makers are likely to make use of the available empirical evidence in order to move beyond ideology [31]. There are at least three different sources of such evidence. First, policy-makers might learn from places where policies on integrated care have already been put into place. Second, policy-makers can make use of the available research literature. Third, policy-makers can gather their own data. Each of these sources of evidence can pose substantial challenges.

First, policy-makers can make use of the experience of other countries that have implemented integrated care policies. This is the domain of policy transfer, a broad concept that can refer to the transfer of: (1) policy goals; (2) policy content; (3) policy instruments; (4) policy programs; (5) institutions; (6) ideologies; (7) ideas and attitudes; and (8) negative lessons^1^ [32]. However, the success or failure of such policy and reform experiments elsewhere may not automatically transfer to the home context. While it may be possible to ascertain that a particular policy was successful in a particular context, it might not always be clear *why* it was successful. Moreover, successfully transferring policy requires not just learning *about* particular policy (such as by reading policy documents), but also learning *from* particular policies (like by talking to the stakeholders involved in the policy’s drafting or implementation). Truly learning from other policies takes time, which is something that many policy-makers deliberating a policy might not have; this all makes learning from elsewhere more difficult than might seem [33].

Secondly, policy-makers can make use of an increasing amount of existing and published evidence from research [13, 14, 34–36]. However, how to translate this knowledge into political action is far from evident. For one, policy-makers often have to make decisions within a limited time frame and, as a result, based on incomplete information [37]. One might, for example, transfer integrated care policy that has been reported as a success. However, the reasons for the success may not necessarily be internal to the network, but might also be external (e.g., relating to the overall health care system or the particular community in which the network operated). These broader external aspects might not be so easily transferable [38]. Furthermore, unlike researchers, policy-makers must take into account potential public support for their measures as well as the political context and reality they operate in. This may substantially limit the amount of reform they can pursue at once [39]. As a result, we know that governments have a strong tendency to adopt incremental policy changes over comprehensive reforms [16, 17].

Apart from the political challenges of turning evidence from research into policy, there are also empirical or methodological challenges. First, there is, as argued above, considerable debate on what constitutes success. Second, while many case studies have been published [39–41], there may be publication or reporting bias, whereby successful networks are more likely to be published about than unsuccessful ones [35]. Third, within the field of policy experimentation there are debates on how best to measure the effect and effectiveness of policy implementation. The challenge here exists in determining the true effect of this experiment or policy. There have been attempts to test the effectiveness of health care policies using randomised controlled trials (RCTs) [42]. This, of course, involves comparing the policy intervention in a particular region or population with a control group without the intervention. In cases of social (health) reform, determining the control group is difficult, and policy research may encounter threats to both internal and external validity [43]. Many other methodologies have been developed and applied in health care policy experimentation [44], such as realist evaluation [45] and contribution analysis [46]. Discussions in these domains are still underway as these methodologies each have their own strengths and challenges.

Third, governments and policy-makers might also gather their own data before implementing policy on a nationwide scale. In 2015, for example, the Belgian federal government and regional governments created a Joint Plan for integrated care for chronically ill patients. For the precise implementation of that plan they selected in 2018 12 smaller scale pilot projects in various parts of Belgium. Knowledge gained through such projects can then provide valuable feedback before the plan is rolled out nationwide. However, such small scale experiments can also entail particular challenges. As an example, one study reported on the challenges encountered by the UK Department of Health and Social Care setting up three smaller researcher-led ‘policy experiments’ [42]. In the end, the Department decided to roll out these pilots studies nationally, but they did so before the final research results were in. This made many of the researchers involved very unhappy, as they wondered to what degree they were ever expected to provide genuine advice based on their expertise. At times, governments may seem preoccupied with generating substantial public support for an ideological position they have already taken.

Despite there being many methodological challenges, these need not necessarily be insurmountable. One way to meet the challenges is for policy-makers who implement policy to also include a mechanism to gather as much data as possible about the workings of the policy and to build in a tool for evaluating the policy after some time. As noted by Campbell, reforms can be considered as akin to (quasi-)experiments that offer unique learning opportunities [43]. Policy-makers can obtain information about how their policy is being implemented, and this information can be fed back into the policy which can then be adjusted, if necessary. This is a potentially realistic way of creating and recreating policy as one goes along [37]. Ideally, these findings could then also be shared so that they can, in turn, inspire others.

#### Resource challenges

Integrated care is often believed to allow for ‘improved efficiency of services, and reduced overall cost’ [12]. However, there is research suggesting that creating integrated care and health care collaborations might actually require a great investment of resources before there is any efficiency pay-off. Three resources are required: (1) expertise, (2) time, and (3) funding.

In terms of expertise, integrated care and health networks differ significantly from more classical hierarchical and market-oriented forms of organization and collaboration [47, 48]. A paper by Keast et al. (2004) suggests that policy-makers may often have an overly classic hierarchical perspective of networks, which shows through in their policy [49]. Successfully harvesting the full potential of integrated care and health networks might, in fact, require a different sort of expertise.

The second resource is time. It has been argued extensively that cooperation and collaboration in networks take time, as they may require the unlearning of previous skills and the learning of new skills [50]. On an operational level, networks might require collaboration in new and smaller groups, which are likely to need to pass through some form of group development (such as the classic four stages of forming, storming, norming, and performing) [51].

The final limitation is funding. It has been shown and demonstrated that, in the short term, promoting collaboration and networks in fact costs money before there is any pay-off [21, 52]. Policy-makers who wish to implement policy need to commit to providing sufficient resources to make that policy work. Weil (2008) wrote the following about states in the US experimenting with health care reforms:

*If we rely upon states to test bold strategies for reform but fail to give them the tools or resources to implement the reforms, we may conclude that certain policies are ineffective despite the fact that under the right circumstances they would perform quite well* [38].

While the aim of integrated care policy might be to save resources, this should be accompanied by a willingness to provide the proper resources in the short term.

### Examples of policy on integrated care

In the previous sections, we have focused on the challenges that arise in drafting and implementing policy on integrated care. In this final section, we would like to briefly discuss three paradigmatic examples of integrated care policy, with a few of their implications.

### Individual patient level integration: the example of Norway

Policy can focus on achieving integration of care for individual patients—for example, by promoting case managers [53] responsible for coordinating individual patients’ care trajectories. Another example is the 3D approach to treating patients with multimorbidity [28]. Such policies or programs work primarily on clinical integration. A good case to consider here is Norway. In 2002, the central Norwegian government took ownership of all public hospitals, creating a highly centralized health care system [53, 54]. In 2001, Norway introduced the right of every patient to have an Individual Care Plan, which names a person who coordinates that patient’s care across the health care system [55, 56]. The focal point in this policy remains strongly with the patient.

Of course, such a policy faces some challenges. For one, since there is less structural collaboration between professionals and institutions, it runs the risk of becoming highly dependent on the ability and willingness of case managers to create such a plan. Research from 2011 suggests that only about 17% of eligible patients actually had an individual plan [55], while other research has even questioned the effectiveness of case managers [57]. The Norwegian government has positively evaluated its policies and will thus maintain its course. However, in view of the challenges mentioned above, the government is planning to make its patient-based approach more structured. In a 2015 white paper, the Norwegian Ministry of Health and Care Services stated that:

*‘The Government is seeking a more structured approach to groups of users based on function and need, independent of diagnosis. A structured approach implies, among other things, a coordinator, use of knowledge-based procedures and checklists, a personalised plan formulated in consultation with the user, and systemic follow-up and evaluation for achieving the objectives set out in the plan. The coordinator leads a team that cooperates with others, preferably across levels, and coordinates the services.’* [58].

### Collaboration for specific conditions or patient groups: the NHS England clinical networks

A second category is policy promoting integration and collaboration for specific conditions [2] or for specific patient groups [11, 12]. A good example here is the National Health Service (NHS) England with its policy of Strategic Clinical Networks. The Scottish Office Health Department has defined these networks as:

*linked groups of health professionals and organisations from primary, secondary, and tertiary care working in a co-ordinated manner, unconstrained by existing professional and [organisational] boundaries to ensure equitable provision of high quality effective services* [38, 66].

Currently, the domains of these NHS England networks are: (1) Cardiovascular care; (2) Maternity, Children, and Young People; (3) Mental Health, Dementia, and Neurological Conditions; and (4) Cancer [59]. By bringing professionals together across organizations, these policies focus on professional and clinical integration. Although more formal than patient-level collaborations, these Networks are not entirely formal or structural. In a *British Medical Journal* editorial, former NHS Confederation Policy Director Nigel Edwards described this as an advantage, and issued a warning against seeing Clinical Networks as ‘the next structural panacea [that would turn] into new NHS organizations’ [38].

Case studies, however, report several challenges to such networks. One study demonstrated how a single English NHS hospital struggled to transform itself from a classic hierarchically structured organization to a more networked community [60]. Another, looking at five NHS cancer-care networks around London, found that four failed to successfully operate as a network [61]. Second, there have been doubts about the effectiveness of promoting interprofessional collaboration. A 2017 Cochrane systematic review looking at the impact of practice-based interventions on improving interprofessional collaboration concluded that:

*Due to the lack of clear evidence, we are uncertain whether the strategies improved patient-assessed quality of care, continuity of care, or collaborative working* [62].

Collaboration between medical professionals from different organizations in a professional network can also raise the issue of conflicting interests, leading to trouble aligning patients’ interests, the interests of the health institutions, and the interests of the clinical network. It has been argued that interprofessional networks function best where they succeed in establishing trust and some sort of group identity [34].

### Institution-wide collaboration: the mandated Belgian hospital networks

The third and final category is policy promoting institution-wide collaboration in broad health care networks. This can include loose networks as well as tighter collaborations, such as health systems in the US [63, 64]. Such policies focus primarily on organizational and systemic integration.

Belgium can be taken as an example here, as the Belgian government has passed legislation mandating every Belgian hospital to enter a larger geographical hospital network [65]. The government has set the maximum number of hospital networks at 25, with the network partners having to agree on the allocation of medical service provision. The government mandates that specialized care cannot be offered at every individual hospital in the network. Compared to networks aimed at specific patient groups or conditions, these institution-wide networks are much broader in scope and are more formalized.

Many challenges remain. For one, the extent to which such collaborations can be successfully *mandated* is questionable [41], as trust between the network members and a willingness to collaborate have been argued to be key ingredients in a network’s success [34]. Moreover, collaboration across the board might prove to be particularly challenging, as different health care institutions often have different interests and moral values, which might not be so easy to align [35].

## Conclusions

It is clear that turning the idea of integrated care into policy is challenging, given the many different types of integration and multiple levels on which it can be achieved. Furthermore, there is ongoing debate about the benefits of integrated care and about the factors that determine its success or failure. When designing and implementing policy, one should always be aware of the political realities in which such policies are drafted, as policy-makers are often required to reach a decision in a limited time and with incomplete information. They also need public or political support for their measures, which means they tend to prefer small changes over large-scale reform.

Nevertheless, despite these challenges, policy will be required to ensure that health care systems provide quality, are financially viable, and are ethically justified. In view of this complexity, we argue that policy-makers should take into account the following:

1. *Reflect on the type and level of integration you want to promote.* As we have argued, integrated care is a broad concept that encompasses various sorts of integration and collaboration. It is necessary as a policy-maker to be aware of the level of integration that you want to achieve as, without proper prior thought, it will be impossible to determine the success of the policy afterwards.
2. *Outcomes: reflect beforehand on what you hope to achieve with integrated care***.** We have argued throughout that integrated care should be seen as a means to a number of possible ends (e.g., economic efficiency or increased quality of care). Knowing what one hopes to achieve by promoting integrated care is crucial to being able to later evaluate the success or failure of the policy. This also allows policy-makers to install a mechanism for evaluation, allowing the health policy to be re-evaluated after a period.
3. *Context: Tailor policy to the particular context in which it will be implemented.* The successful integration of a given policy in a particular health care context might not be automatically transferrable to another health care context. Policy-makers should critically assess the available scientific literature and look at examples of places where comparable policies have been implemented. One cannot simply copy policy from somewhere else and expect it to work. Policy-makers should preferably not only learn *about* what happened in other places, but instead learn *from* other places.
4. *Resources: be committed to investing the resources needed to genuinely run and evaluate a policy*. Research shows how successfully promoting integration may require resources such as time, expertise, and funding. As we have argued, policy-makers who fail to invest the necessary amount of money might afterwards incorrectly conclude that a particular policy implementation does not work.
5. *Provide the stakeholders with sufficient freedom and autonomy.* Research shows that successful integration cannot be fully mandated, and requires a willingness from stakeholders and a relationship of trust between them.
6. *Consider beforehand how the actual implementation of the policy can be evaluated*: Despite there being empirical challenges, a lot can be learned from the example of other countries and the experiences of other policy makers. There is also the option of setting up pilot projects or policy experiments to gather relevant feedback. Finally, policy makers should also consider the installation and use of feedback mechanisms to gain insight into the implementation of policy once it is underway.

## Data Availability

Not applicable.

## Abbreviations

GDPR: General Data Protection Regulation
IHI: American Institute of Health Care Improvement
NHS: National Health Service
RCT: Randomised controlled trial

## Footnotes

1 This is an interesting category that refers to policy-makers using foreign examples to learn what *not* to do.
